# Yoga and Telomeres: A Path to Cellular Longevity?

**DOI:** 10.7759/cureus.74552

**Published:** 2024-11-27

**Authors:** Selvaraj Giridharan

**Affiliations:** 1 Oncology, Tawam Hospital, Al Ain, ARE

**Keywords:** longevity, meditation, telomerase, telomeres, yoga

## Abstract

Telomeres, which protect the chromosomal ends, are vital for cellular senescence and health. Telomere shortening, often due to stress, inflammation, and oxidative damage, is linked to age-related diseases such as cancer, cardiovascular issues, and neurodegeneration. Evidence suggests that meditation may affect telomere dynamics by reducing stress and inflammation and improving emotional regulation. Clinical trials have demonstrated that the effectiveness of these practices in increasing telomerase activity and maintaining telomere length varies by type, intensity, and duration of the practice. Yoga and meditation boost cellular resilience by lowering stress, inflammation, and oxidative damage and enhancing neuroendocrine regulation. Despite promising results, study design variability and limited long-term data require further research. Future studies should identify the most effective components, dose-response relationships, and long-term effects across populations. Increasing evidence suggests that yoga and meditation could be key preventive and therapeutic strategies to improve cellular health and longevity.

## Editorial

Introduction

Telomeres have attracted significant attention for elucidating the mechanisms of aging and health. These repetitive nucleotide sequences safeguard chromosomal integrity and play a pivotal role in cellular senescence and aging. However, telomere shortening, which is accelerated by stress, inflammation, and oxidative damage, is linked to age-related diseases, such as cardiovascular disorders, cancer, and neurodegenerative conditions​ [1]. Emerging research has emphasized the potential of lifestyle interventions, particularly yoga and meditation, to counteract telomere attrition and promote cellular health. These non-invasive, holistic practices modulate biological processes such as stress reduction, emotional regulation, and inflammation control [2]. They hold promise in maintaining telomere length and enhancing telomerase activity, the enzyme responsible for telomere repair [3]. Observational studies and clinical trials have shown significant enhancements in telomerase activity and modest preservation of telomere length among practitioners [4]. This editorial explores the intersection of yoga, meditation, and telomere biology, highlighting its potential as a tool for promoting cellular resilience and longevity.

Telomeres, telomerase, and the role of stress in cellular aging

Telomeres, which are essential for genomic stability, naturally shorten with cell division, eventually triggering senescence or apoptosis. This attrition is exacerbated by oxidative stress, chronic inflammation, and environmental factors, which accelerate aging and increase vulnerability to disease [2]. Telomerase partially counters this process by adding telomeric repeats; however, its activity is limited in most somatic cells, especially under chronic stress​ [1,3]. Psychological stress, a key driver of telomere dynamics, increases cortisol levels, promotes systemic inflammation, and accelerates oxidative damage. Interventions such as yoga and meditation mitigate these effects by reducing stress biomarkers, lowering inflammatory markers, and enhancing telomerase activity [4]. Therefore, these practices have emerged as integral strategies for telomere preservation and overall cellular health.

Clinical evidence on telomere dynamics

Several clinical trials have explored the effects of yoga and meditation on telomere length and telomerase activity, providing insights into their potential roles in mitigating cellular aging [5-10]. A randomized controlled trial by Sharma et al. investigated the effects of a 12-week yoga-based lifestyle intervention (YBLI) on telomere length and telomerase activity in obese individuals. Participants practicing yoga, including asanas, pranayama, and meditation, demonstrated significant improvements in telomere length after two weeks compared to standard care, although the differences were not maintained at 12 weeks [5]. Le Nguyen et al. conducted a 12-week trial comparing mindfulness meditation (MM), loving-kindness meditation (LKM), and a control group in midlife adults. The study found that while telomere length decreased in the MM and control groups, the LKM group experienced less attrition, underscoring the unique ability of LKM to buffer telomere shortening [6]. Jacobs et al. examined the effects of a three-month meditation retreat on telomerase activity. Participants who meditated intensively for six hours daily showed significantly greater telomerase activity than controls. Psychological improvements, such as increased perceived control and decreased neuroticism, mediate this effect, linking positive emotional regulation to cellular health [7].

Carlson et al. evaluated the impact of Mindfulness-Based Cancer Recovery (MBCR) and Supportive Expressive Therapy (SET) on telomere length in distressed breast cancer survivors. While neither intervention led to significant differences in telomere length, the combined intervention group showed a trend toward telomere length maintenance, in contrast to the decrease in the control group. This finding supports the potential of mindfulness practices to preserve telomere length under chronic stress conditions [8]. Lavretsky et al. studied the effects of a brief daily yogic meditation program, Kirtan Kriya, on family dementia caregivers. Participants practicing meditation for 12 minutes daily over eight weeks showed a 43% improvement in telomerase activity, along with better mental health and cognitive outcomes compared to controls. These findings reinforce the notion that even brief and consistent meditation practices can significantly influence cellular aging [9]. A trial by Puhlmann et al. explored the association between leukocyte telomere length (LTL) changes and cortical thickness in healthy adults undergoing mindfulness training. While no direct impact of mindfulness on LTL was observed, LTL changes correlated with brain structure changes, suggesting potential indirect effects of mindfulness on cellular and neurological health​ [10].

Mechanisms of action

The mechanisms by which yoga and meditation influence telomere dynamics are multifaceted and involve both direct biological effects and indirect pathways mediated through psychological and behavioral changes. These mechanisms collectively contribute to the maintenance of telomere length, enhancement of telomerase activity, and reduction in cellular aging.

Reduction of Psychological Stress

Chronic stress is a well-documented accelerator of telomere shortening mediated by elevated cortisol levels and heightened inflammatory responses. Yoga and meditation practices were associated with significant reductions in psychological stress, leading to lower cortisol levels and improved emotional regulation​ [1]. For instance, studies on caregivers and cancer survivors have shown that meditation reduces stress biomarkers while preserving telomere length or increasing telomerase activity​ [9].

Anti-inflammatory Effects

Inflammation, driven by elevated cytokines, such as tumor necrosis factor-alpha (TNF-α) and interleukin-6 (IL-6), contributes to telomere attrition. Mindfulness-based interventions have been shown to reduce systemic inflammation, which may partially mediate their protective effects on telomeres [1,2]. Evidence from meditation trials, including those by Lavretsky et al. and Jacobs et al., demonstrates significant reductions in inflammatory markers alongside improvements in telomerase activity [7,9].

Reduction of Oxidative Stress

Oxidative stress, another key driver of telomere shortening, results from an imbalance between reactive oxygen species (ROS) and the body's antioxidant defenses. Yoga and meditation have been shown to enhance antioxidant activity, potentially mitigating oxidative damage to the telomeres. This pathway has been particularly highlighted in trials involving individuals with obesity or chronic stress [5].

Improvement in Psychosocial Factors

Psychological traits, such as perceived control, resilience, and mindfulness, have been linked to telomere biology. The Jacobs et al. trial demonstrated that improvements in perceived control and reductions in neuroticism mediated the effects of meditation on telomerase activity​ [7]. Similarly, the LKM trial indicated that cultivating positive emotions might buffer telomere attrition [6].

Enhanced Neuroendocrine Regulation

Yoga and meditation may exert their effects through the modulation of the hypothalamic-pituitary-adrenal (HPA) axis, a key regulator of stress responses. These practices create a physiological environment conducive to telomere preservation​ [4].

Behavioral and Lifestyle Changes

Long-term yoga and meditation practitioners often adopt healthier behaviors, including an improved diet, increased physical activity, and better sleep patterns. These lifestyle factors synergize with the direct effects of mindfulness practices to support telomere health​ [3].

Understanding the impact of yoga and meditation on telomere biology is vital for optimizing these practices for improving cellular health. Future research should pinpoint specific elements such as asana, pranayama, and mindfulness that most affect telomere preservation and explore dose-response relationships to identify the minimum effective duration and intensity. Examining the long-term sustainability of these effects across diverse populations will strengthen the evidence base, enabling the development of tailored evidence-based interventions to improve cellular longevity and overall well-being.

Challenges, limitations, and future directions

Yoga and meditation benefit telomere biology; however, several challenges and limitations must be addressed for their effective application in clinical and public health contexts. Studies vary widely in sample size, demographics, and intervention protocols, complicating generalization across diverse populations with unique stress profiles and baseline telomere characteristics [2]. Different yoga and meditation practices, such as mindfulness, LKM, asanas, and pranayama, exert varying effects on telomere biology, with some practices, such as LKM, showing unique protective effects against telomere attrition [6].

Accurate measurement of telomere length and telomerase activity requires standardized laboratory protocols, and variability in measurement techniques can cause inconsistencies in findings [1,4]. Longitudinal assessments are necessary owing to the dynamic nature of telomerase activity. However, most studies to date have assessed outcomes over short periods, thus limiting insights into long-term benefits. Prospective studies that track changes over time are needed to establish lasting effects on cellular aging [2]. Additionally, behavioral changes accompanying yoga and meditation complicate the isolation of their independent effects on telomere dynamics [3].

Large multicenter randomized controlled trials are essential to corroborate the existing findings and refine the intervention protocols. However, such studies have encountered substantial practical and financial challenges. Multicenter trials require coordination across multiple research sites, thereby increasing the logistical complexity. Furthermore, interventions involving yoga and meditation require skilled instructors, tailored participant guidance, and long-term follow-up, all of which contribute to increased costs. The requirement for repeated telomere and telomerase measurements using advanced techniques further elevates expenses. Funding constraints frequently limit the feasibility of adequately powered studies, resulting in small-scale trials with limited statistical robustness.

Future research should aim to optimize study designs by exploring cost-effective strategies such as incorporating remote interventions, utilizing simplified biomarkers, or focusing on high-risk populations where telomere-related benefits may be most pronounced. Elucidating the molecular mechanisms linking yoga and meditation to telomere biology, such as epigenetic modifications, oxidative stress reduction, and immune system modulation, is imperative. Additionally, evaluating the cost-effectiveness of implementing yoga and meditation programs in clinical and community settings is crucial for expanding accessibility and acceptance.

Conclusion

Research in yoga and meditation is crucial for enhancing cellular health and longevity in mind-body medicine, particularly through the study of telomeres and telomerase, which are markers of cellular aging. These practices have been shown to reduce stress and inflammation, boost telomerase activity, and maintain the telomere length. However, inconsistencies in study design, participant demographics, and intervention methods call for more standardized research efforts. Longitudinal studies are essential for confirming their long-term effects on cellular aging. Integrating yoga and meditation into preventive and therapeutic regimens could naturally reduce stress and its effects on cellular health, thereby improving psychological well-being, emotional resilience, and quality of life (QoL). Advancing research will pave the way for tailored interventions that enhance health span and lifespan, positioning yoga and meditation as key to promoting cellular longevity and better health.
